# Prediction of the general transcription factors associated with RNA polymerase II in *Plasmodium falciparum*: conserved features and differences relative to other eukaryotes

**DOI:** 10.1186/1471-2164-6-100

**Published:** 2005-07-23

**Authors:** Isabelle Callebaut, Karine Prat, Edwige Meurice, Jean-Paul Mornon, Stanislas Tomavo

**Affiliations:** 1 Centre National de la Recherche Scientifique CNRS UMR7590, Universités Paris 6 et Paris 7, Département de Biologie Structurale, IMPMC, case 115, 4 place Jussieu, 75252 Paris Cedex 05, France; 2 Centre National de la Recherche Scientifique CNRS UMR 8576, Université des Sciences et Technologies de Lille, Equipe de Parasitologie Moléculaire, Laboratoire de Chimie Biologique, UGSF, Bâtiment C9, 59655 Villeneuve d'Ascq, France

## Abstract

**Background:**

To date, only a few transcription factors have been identified in the genome of the parasite *Plasmodium falciparum*, the causative agent of malaria. Moreover, no detailed molecular analysis of its basal transcription machinery, which is otherwise well-conserved in the crown group of eukaryotes, has yet been reported. In this study, we have used a combination of sensitive sequence analysis methods to predict the existence of several parasite encoded general transcription factors associated with RNA polymerase II.

**Results:**

Several orthologs of general transcription factors associated with RNA polymerase II can be predicted among the hypothetical proteins of the *P. falciparum *genome using the two-dimensional Hydrophobic Cluster Analysis (HCA) together with profile-based search methods (PSI-BLAST). These predicted orthologous genes encoding putative transcription factors include the large subunit of TFIIA and two candidates for its small subunit, the TFIIE β-subunit, which would associate with the previously known TFIIE α-subunit, the TFIIF β-subunit, as well as the p62/TFB1 subunit of the TFIIH core. Within TFIID, the putative orthologs of TAF1, TAF2, TAF7 and TAF10 were also predicted. However, no candidates for TAFs with classical histone fold domain (HFD) were found, suggesting an unusual architecture of TFIID complex of RNA polymerase II in the parasite.

**Conclusion:**

Taken together, these results suggest that more general transcription factors may be present in the *P. falciparum *proteome than initially thought. The prediction of these orthologous general transcription factors opens the way for further studies dealing with transcriptional regulation in *P. falciparum*. These alternative and sensitive sequence analysis methods can help to identify candidates for other transcriptional regulatory factors in *P. falciparum*. They will also facilitate the prediction of biological functions for several orphan proteins from other apicomplexan parasites such as *Toxoplasma gondii*, *Cryptosporidium parvum *and *Eimeria*.

## Background

Each year 300–500 million people suffer from malaria while 1.5 to 2 million, mostly children, die as a result of the infection (Global Health Council, 2003). The lethal form of human malaria is caused by the infection with the obligate intracellular protozoan parasite *Plasmodium falciparum*, which displays a developmental life cycle alternating between a vertebrate and an invertebrate host. Infection by the sporozoite form of the parasite occurs after the female *Anopheles *mosquito's bite. The parasite then enters hepatocytes and multiplies by an asexual division process named schizogony. The resulting merozoites then invade erythrocytes and the parasite goes through a series of morphological changes upon massive rounds of asexual division (ring, trophozoite, schizonte and merozoite). The intermittent fevers, characteristic of malaria infection, are attributed to cycles of erythrocyte invasion, asexual reproduction by schizogony, and release of asexual parasites (merozoites) after rupture of infected red blood cells. For completion of the host-vector cycle, some intra-erythrocytic asexual forms do not undergo schizogony but transform into sexually dimorphic male and female gametocytes upon differentiation. Gametocytes are taken into the mosquito's midgut during a blood meal and complete their sexual development to gametes which will fuse to form a motile zygote named the ookinete. The ookinete grows into an oocyst, dividing into numerous sporozoites that will invade the salivary glands of the mosquito ready for a new cycle of infection [1].

During the complex life cycle of *P. falciparum *which takes place in both a vertebrate and an invertebrate host, the intracellular development of the different asexual and sexual stages proceeds through a dynamic and multistep process for which the parasite has evolved complex molecular strategies. Several pioneering studies have previously demonstrated that transcriptional regulations are involved in the control of gene expression in the various *P. falciparum *life cycle forms [2-6]. The recent completion of the full genome sequence of *P. falciparum *has been useful in studying the global and complex gene expression patterns using microarrays and proteomic approaches. Indeed, these studies suggested that there is a coordinated program of gene expression during the intra-erythrocytic development of the parasite. Microarray data have revealed a sequential expression of transcripts in which messenger RNAs involved in protein synthesis peak at first, followed by metabolism-related genes, then adhesion/invasion genes, and lastly protein kinases [7-10]. Global proteome analysis of sporozoites, merozoites, trophozoites, and gametocytes using tandem mass spectrometry analysis have been used to show that many co-expressed proteins are encoded by genes that are clustered on certain chromosomes [11,12]. These recent studies on gene expression also show that transcription of multiple genes may be achieved by a single developmental induction event resulting in a cascade of gene expressions. This further suggests that only a few specific transcription factors may be required [10]. Nevertheless, it has been established that the gene structure of *P. falciparum *is similar to that of other eukaryotes [13,14], with the common features including the monocistronically transcribed genes, the presence of 5' and 3' untranslated regions, introns, promoter regions and probably the myriad of transcription factors that are involved in eukaryotic gene expression in general.

Transcription in eukaryotic structural genes requires the assembly of RNA polymerase II (RNAP II) and the general transcription factors (GTFs) on the promoter to form a pre-initiation complex. These basic factors include RNA polymerase II itself and at least six GTFs: TFIIA, TFIIB, TFIID, TFIIE, TFIIF and TFIIH, most of which are themselves multiprotein complexes [15,16]. While the RNA polymerase I, II, III and TATA-binding protein [17-21] have been described in *P. falciparum*, the elucidation of the mechanisms involved in transcriptional regulation in the parasite is still challenging. For example, the identification of orthologous proteins including the general transcription factors (GTFs) involved in the RNAP II transcription machinery remains elusive. Therefore, the composition and nature of the highly conserved general transcription factors associated with the RNAP II are presently unknown in *P. falciparum*. In contrast to most eukaryotic genomes, the extensive analysis of the *P. falciparum *genome has only revealed a few general transcription factors, like TBP and TFIIB [22]. More recently, Coulson et al. [23] have utilized profile-Hidden Markov Models (HMMs) of transcriptional regulators and found a relatively low number of malarial transcription-associated proteins (TAPs) including the general transcription factors associated with RNAP II. Only TFIIB, TFIIEα and a few components of TFIIH were identified in *P. falciparum*. In addition, no homolog of the RNAP II-associated TFIID complex, which is essential for the basal transcription in eukaryotes, was found, except the TATA-binding protein (TBP) [23]. Therefore, it has been suggested that only a few specific transcription factors may be required for transcription regulation in the parasite. However, the parasite protein levels may also be primarily determined by posttranscriptional mechanisms [9,10,23]. The high proportion of orphans in the *Plasmodium *genome relative to other organisms (~60% ORFs which have no match with any known sequences [24]) suggests that the paucity of recognizable orthologous GTFs associated with RNAP II in *P. falciparum *may be explained in a different way. As ORF, gene and function predictions have been performed in a similar way in *Plasmodium *and in other sequenced genomes (such as various predictive tools trained on the *Plasmodium *sequences; BLAST with default parameters [24,25]), two hypotheses can be raised. First, it is possible that the parasite proteins have structurally evolved beyond the point where they cannot be identified by simple similarity searches [23,26]. Second, the extraordinary bias toward A+T richness (80%) in nucleotide composition of the parasite, may introduce large changes in both DNA and amino acid sequences which may affect the search procedures. This is particularly striking with an overall high A+T nucleotide content in protein-coding regions, leading to a remarkable bias toward the presence of stretches composed of a few amino acids only. Therefore, it is likely that a substantial number of the unusually high proportion of malarial orphan proteins with no predictable function may actually correspond to «hidden» orthologues. Interestingly, we and others ([22,27]; our unpublished results) have observed that there is a strong selection against low complexity inserts within core secondary elements of secondary structures of *P. falciparum *proteins. The low complexity sequences are mostly located between two adjacent globular domains and only infrequently invade globular domains.

In the present study, we postulated that the hydrophobic cores of globular domains in functional proteins of *P. falciparum *should be largely conserved. Consequently, these hydrophobic cores could be identified using appropriate tools involving the analysis of the secondary structure, which is often much more conserved than the primary structure [28]. We have developed and applied a two-dimensional approach of sequence analysis, called Hydrophobic Cluster Analysis (HCA), which has been useful for the prediction of orthologous proteins in different eukaryotic lineages [29,30]. HCA is based on the physico-chemical and topological properties underlying the fold of globular domains. It allows a direct access to the gravity centers of regular secondary structures (RSSs). This information can be used to pick up hidden relationships within non-significant results provided by standard similarity search methods, based on literal approaches. Indeed, the positions of hydrophobic clusters defined using HCA, which distinguish from simple binary patterns, mainly correspond to those of regular secondary structures [31,32]. Importantly, HCA is not sensitive to gaps, even large, the handling of which is one of the main obstacles of conventional sequence comparison methods. The distribution of the secondary structures also indicates the limits of structured domains. This information can help the computational analysis, in particular for *P. falciparum *sequences for which low complexity regions often disturb standard similarity searches. Using the HCA methodology in combination with standard similarity search methods, we have explored the *P. falciparum *sequences for the presence of subunits of the basal transcription factors and cofactors associated with RNA polymerase II (RNAP II). Our data suggested that several orphan proteins of *P. falciparum *can be predicted as general transcription factors involved in the parasite RNAP II transcription machinery.

## Results

**Figure 1:**
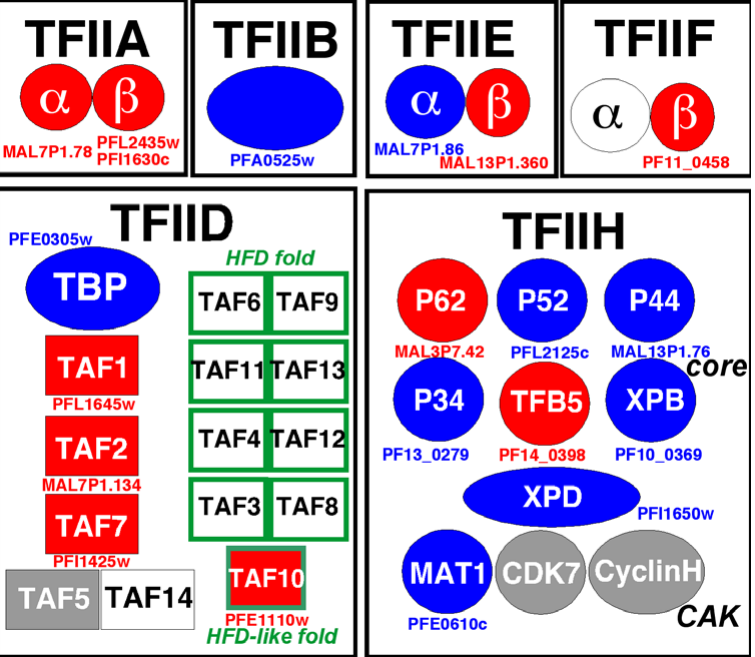
Schematic view of the predicted general transcription factors associated with RNA polymerase II in *Plasmodium falciparum*. Components which have been predicted in previous studies and in the present analysis, are displayed respectively in blue and in red. The components which have not been predicted from sequence analyses are shown in grey and white. Grey boxes indicate components for which potential candidates exist, but which cannot be discriminated from sequence analysis alone, due to the absence of specific domains. Green boxes indicate the HFD-containing TAF pairs which have not been identified in *Plasmodium falciparum*.

We have collected protein sequences from the different subunits of the basal transcription factors and cofactors in different genomes. The *Homo sapiens *and *Saccharomyces cerevisiae *sequences used are listed in Table 1. These sequences were used as queries for PSI-BLAST searches within the non-redundant database (nr) at NCBI. This search leads to the construction of profiles specific to each protein or protein domain. All *P. falciparum *sequences predicted in this study are underlined in Table 1 and shown in red in Fig. 1. Several *P. falciparum *sequences were easily identified as significantly matching with some subunits of known basal transcription factors of the complex RNAP II machinery. It appears that only a few of these putative parasite transcription factors such as TFIIB, TFIIE-α and several components of TFIIH, correspond to those reported elsewhere [23] (non-underlined sequences in Table 1, indicated in blue in Fig. 1). However, in several cases, the two-dimensional analysis provided by HCA led to extend the similarity outside of the limits initially reported by PSI-BLAST (hits indicated with the symbol "+" in Table 1). When no significant similarity was highlighted in the PSI-BLAST data, marginal similarities (*Expected values *above the threshold value) were investigated using HCA. This led to the prediction of several hypothetical protein or orphans as novel, potential orthologous basal transcription factors and cofactors associated with the RNAP II machinery in *P. falciparum *(sequences with an asterisk (*) in Table 1, indicated in red in Fig. 1).

**Table 1:** General transcription factors predicted in *Plasmodium falciparum*

**Factors**	***H. sapiens***	***S. cerevisiae***	***P. falciparum***	**Nuclear signals prediction**		**Expression pattern**	
				
				**NLS**	**NES**	**Micro-array**	**Proteomic data**
**TFIIA α**	P52655	P32773 (TOA1)	**MAL7P1.78 +**	-	-	G	-
**TFIIA β**							
**TFIIA γ**	P52657	P32774 (TOA2)	PFL2435w *+	-	-	T,Sc	-
			PFI1630 *	+	-	G	-
**TFIIB**	Q00403	P29055	PFA0525w	-	-	All stages	-
**TFIID TBP**	P20226	P13393	PFE0305w	-	-	R	-
**TFIID TAF1**	P21675 (TAF250)	P46677 (TAF145)	**PFL1645w**	+	-	S,LT	S,G
**TFIID TAF2**	*gi:4507347 *(TAF150)	P23255 (TAF150)	**MAL7P1.134**	+	+	R,T	S
**TFIID TAF5**	Q15542 (TAF100)	P38129 (TAF90)	?				
**TFIID TAF7**	Q15545 (TAF55)	Q05021 (TAF67)	**PFI1425w**	+	+	R,S	-
**TFIID TAF14**	P42568 (ENL/AF-9)	P35189 (TAF30)	?				
**TFIID TAF4**	O00268 (TAF135)	P50105 (TAF48)	?				
**TFIID TAF12**	Q16514 (TAF20)	Q03761 (TAF68/61)	?				
**TFIID TAF6**	P49848 (TAF80)	P53040 (TAF60)	?				
**TFIID TAF9**	Q16594 (TAF31)	Q05027 (TAF17)	?				
**TFIID TAF11**	Q15544 (TAF28)	Q04226 (TAF40)	?				
**TFIID TAF13**	Q15543 (TAF18)	P11747 (TAF19)	?				
**TFIID TAF3**	*gi:13374079 *(TAF140)	Q12297 (TAF47)	?				
**TFIID TAF8**	*gi:31323620 *(TAF43)	Q03750 (TAF65)	?				
**TFIID TAF10**	Q12962 (TAF30)	Q12030 (TAF25)	**PFE1110w**	-	+	R, Sc	-
**TFIIE α**	P29083	P36100	MAL7P1.86 +	+	+	Sc	-
**TFIIE β**	P29084	P36145	MAL13P1.360 *	+	-	ND	-
**TFIIF α**	P35269 (RAP74)	P41895 (Tfg1)	?				
**TFIIF β**	P13984 (RAP30)	P41896(Tfg2)	PF11_0458 *	-	+	R,G	-
**TFIIH core p62/TFB1**	P32780 (p62)	P32776 (TFB1)	MAL3P7.42 *+ (Chr3.phat_258)	+	+	R,T	-
**TFIIH core p52/TFB2**	Q92759 (p52)	*gi:6325135 *(TFB2)	PFL2125c	+	+	R,T,Sc	-
**TFIIH core p44/SSL1**	Q13888 (p44)	Q04673 (SSL1)	MAL13P1.76	+	+	R,T	-
**TFIIH core p34/TFB4**	Q13889 (p34)	*Gi:6325313*	PF13_0279	-	+	T	-
**TFIIH core TFB5**	*Gi:55665883*	*Gi:13129164*	**PF14_0398**	-	-	R, T, G	-
**TFIIH core XPB/SSL2-RAD25**	P19447 (XPB)	Q00578 (SSL2/RAD25)	PF10_0369	+	-	G	S
**TFIIH XPD/RAD3**	P18074 (XPD)	P06839 (RAD3)	PFI1650w	+	+	R,T,G	G
**TFIIH CAK MAT1/TFB3**	P51948 (MAT1)	*Gi:6320668 *(TFB3)	PFE0610c	+	-	R,T	-
**TFIIH CAK Cdk7/KIN28**	P50613 (CDK7)	P06242 (KIN28)	? $				
**TFIIH CAK Cyclin H/CCL1**	P51946 (cyclin H)	P37366 (CCL1)	? $				

The general transcription factors which were identified in this report are underlined and shown in bold; * and + indicate similarities which were identified in this report after assessing PSI-BLAST marginal similarities at the sequence 2D level or after extending the comparison at the 2D level outside the limits primarily defined by PSI-BLAST, respectively. The Swiss-Prot accession numbers are given for the human and yeast sequences. When a Swiss-Prot identifier is not available, the genbank identifier (gi) is indicated in italics instead. The references of the human TAF3 and TAF8 sequences can be found in [50] and [100], respectively. $ : see text for comments on these putative homologues of Cdk7/KIN28 and cyclin H/CCL1. The presence of nuclear localization sequences (NLS 102), nuclear export sequences (NES [103]), and the expression of these predicted general transcription factors using microarray and proteomics on different parasite stages ([9–11, 89]) are indicated. G: gametocyte, T: trophozoite, LT: late trophozoite, Sc: schizonte, R: ring, (-): absent, (+): present, (?): cannot be found.

In all cases with marginal similarities (PSI-BLAST E-values > 0.005), the alignment with the candidate hypothetical protein has low Expected E-values, proximal to the threshold value. These values are lower than those observed for alignments with other *P. falciparum *hypothetical proteins. However, other potential candidates (which have higher Expected E-value) were carefully checked by HCA for similarities that might be supported at the 2D level.

Except one of the hypothetical proteins, all predicted proteins here as general transcription factors in *P. falciparum *have corresponding sequences in other *Plasmodium *species (Table 2), with identity levels above 50%. TAF10 is the only exception as it is apparently not present in *P. yoelii *(see Discussion). This overall conservation provides support for the prediction of the *P. falciparum *hypothetical proteins listed in Tables 1 and 2 as potential important components of the basic transcription machinery.

**Table 2:** General transcription factors predicted in this study in four *Plasmodium *species

	***P. falciparum***	***P. yoelii yoelii***	***P. chabaudii***	***P. berghei***	**Reciprocal Search**
TFIIA large subunit	MAL7P1.78 *197 aa*	PY01022 57% N-ter 133 aa*	PC302380.00.0 57% N-ter 133 aa*	PB000347.02.0 59% N-ter 133 aa*	+
TFIIA small subunit	PFL2435w 131 aa	chrPyl_02265-4-2031-1630 *71% tl 134 aa*	PC403116.00.0 68% 43 aa (partim)	PB101071.00.0 63% 36 aa (partim)	*B: A.thaliana (gi 1429228)*
	PFI1630c *184 aa°*	PY01831 *51%tl 200 aa*	PC000365.00.0 *80% tl 105 aa*	PB001668.02.0 *95% tl 200 aa*	*B: G. theta (gi 4583664)*
TFIID Taf1	PFL1645w *3896 aa*	PY03752 *65% $ 3182 aa*	PC000201.00.0 *64% $ 1254 aa*	PB000870.00.0 *64% $ 843 aa*	+
TFIID Taf2	MAL7P1.134 *3351 aa*	PY03343 *80% $ 1684 aa*	PC000872.02.0 *80% $ 1353 aa*	PB000540.02.0 *78% $ 926 aa*	+
TFIID Taf7	PFI1425w *397aa*	PY04173 *58% tl 321 aa*	PC000532.04.0 *56% tl 387 aa*	PB000149.02.0 54% 325 aa	+
TFIID Taf10	PFE1110w *116 aa*	?	genomic	PB108412.00.0 *51% tl 93aa*	*B: O.sativa (gi 50726230)*
TFIIE α-subunit	MAL7P1.86 *400 aa*	PY00824 *57% tl 369 aa*	PC000361.01.0 *64% tl 386aa*	PB000518.01.0 *64% tl 381 aa*	+
TFIIE β-subunit	MAL13P1.360 *542 aa*	PY01317 *53% $ 2329 aa*	PC103304.00.0 *81% $ 207 aa*	PB100065.00.0 *54% $ 548 aa*	*B: S.cerevisiae (sp P36145)*
TFIIF β-subunit	PF11_0458 *317 aa*	PY03467 *60% tl 310 aa*	?	PB000215.00.0 60% tl 175 aa*	*B: C.parvum (gi 46228562)*
TFIIH P62	MAL3P7.42 *670 aa*	PY00359 *59% tl 674 aa*	PC000077.04.0 *62% tl 682 aa*	PB000867.00.0 *71% tl 343 aa*	-
TFIIH TFB5	PF14_0398 *67aa*	chrPyl_00238-4-3595-3377 *92% tl 73 aa*	Pc_1897-6-1673-1455 *92% tl 73 aa*	PB000215.03.0 91% tl *67 aa*	+

The general transcription factors predicted in this study are listed with the corresponding sequences in *Plasmodium yoelii yoelii*, *Plasmodium chabaudii and Plasmodium berghei*. Reciprocal searches were performed using each predicted transcription factor, leading to the finding of similarities with the corresponding subunit in the crown group eukaryotes (see comments in the text; + stands for significant, B stands for background (E value >0.005)). The percentages of identity with the *P. falciparum *sequences are indicated (*tl *stands for calculated on the total length of the *Plasmodium *sequences), as well as the lengths of the considered protein sequences. $ : calculated on the similarity regions discussed in the text *: likely uncomplete sequence; °: presently corrected sequence; genomic: found in the genomic sequences, using TBASTN. 2.1.2.

Reciprocal searches were carried out for all the predicted GTF components. In most cases (indicated with a "+" in Table 2), these led to the retrieval, with significant E-values, of the corresponding sequences in other eukaryotes. The reciprocal searches were often conducted using as a probe the similarity region, excluding low complexity regions that are abundant in *P. falciparum *sequences. However, the profiles deduced from the *P. falciparum *sequences are generally less informative than those constructed using as probes the human or yeast sequences. As a consequence, such reciprocal searches resulted, in a few cases, in the retrieval of the corresponding sequences in other eukaryotes with marginal, but low expected E-values (just above the threshold E-value). It should be noticed that the sequences of another apicomplexan parasite, *Cryptosporidium parvum*, often constituted the link between *Plasmodium *and the crown group eukaryotes.

Additional support for our predictions also comes from other data, such as the prediction of nuclear localisation and nuclear export signals (NLS and NES), as well as the analysis of expression patterns (Table 1). However, it should be mentioned that nuclear factors do not always require the presence of NLS or NES for their targeting into the nucleus. For instance, it has recently been described that the nuclear transport of human TAF10, which lacks both NLS and NES, is mediated by its interacting partners, which contain the nuclear targeting signals [33].

Throughout this study, we decided to designate the putative transcription factor, _*Pf*_*TFIIA *for *P. falciparum *ortholog of higher eukaryote TFIIA. The same nomenclature will be used for the other basal transcription factors and cofactors identified here.

### _Pf_TFIIA

The TFIIA proteins form a ternary complex with TBP and DNA. It stabilizes the TBP-DNA binding and promotes the binding of TFIID complex to DNA. Yeast TFIIA is composed of two subunits (TOA1 and TOA2), which can each be divided in two parts, a N-terminal helical region and a beta-strand containing C-terminal region. The N-terminal regions of the two subunits form together a four-helix bundle, whereas the two C-terminal ones fold as a six-stranded beta-barrel contacting TBP-DNA [34,35]. The human TFIIA homologue is made of three polypeptide chainsα/β (large subunit encoded by a single chain, which is post-translationally processed) and γ (small subunit) [36].

**Figure 2:**
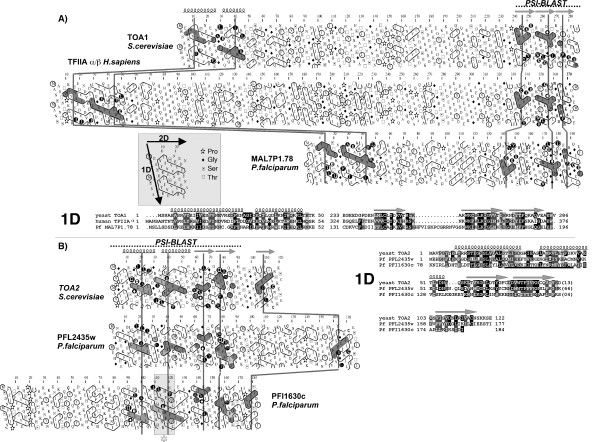
Comparison of the HCA plots of TFIIA large subunit (TOA1/TFIIA α/β panel A) and small subunit (TOA2/TFIIA γ panel B) subunits from different species, highlighting the conservation of the hydrophobic core of the domains constituting the two proteins in the hypothetical proteins from *Plasmodium falciparum*. The sequences are shown on a duplicated α-helical net, in which strong hydrophobic amino acids (VILFMYW) are circled. These form clusters, which mainly correspond to the internal faces of regular secondary structures (α-helices and β-strands). The way to read the sequence and special symbols is indicated in the inset. The regions initially detected by PSI-BLAST (either with significant (TOA1/TFIIA α) or marginal *E-value *(TOA2/TFIIA γ) are indicated with a dotted line. Cluster similarities are shaded in grey, identities are shown in white on a black background. Despite low level of sequence identity, hydrophobic clusters are well conserved, supporting the presence of a common fold. The deduced 1D alignment is shown at the bottom and at right. The positions of regular secondary structures, as observed from experimental data (pdb 1nh2) are shown up to the HCA plot. Two hypothetical proteins from *Plasmodium falciparum *share significant similarities with the small subunit of TFIIA (bottom panel) and are thus _Pf_TFIIA small subunit candidates. The similarity with PFI1630c was revealed using its homolog sequence in *Plasmodium yoelii *(see text). This sequence was missed during our first PSI-BLAST search because of an error in the intron prediction. This novel ortholog has been found through HCA sequence comparison of the translated DNA sequences (the boxed and underlined sequences in the HCA plot and 1D alignment, respectively, correspond to the sequence which was first included in an intron, as predicted automatically from genome data).

The *P. falciparum *orthologous TFIIA large subunit was easily identified using the yeast TOA1 sequence. We found that the C-terminal part of this first subunit TOA1 sequence (aa 214 to 285) can be aligned with a significant PSI-BLAST E-value (E-value 3 10^-4 ^by iteration 2) with the C-terminal part of the hypothetical protein MAL7P1.78 from *P. falciparum *(Fig. 2, panel A). Although no similarity was highlighted by PSI-BLAST with the N-terminal part of the proteins, HCA indicates the presence of large helices in the N-terminal part of the malarian protein, which can be aligned with those of the yeast and human sequences (Fig. 2, panel A). This N-terminal sequence similarity could not be detected, even in the background noise, using the first 100 amino acids of either TFIIA α (yeast and human sequences) or MAL7P1.78 as queries in PSI-BLAST searches. Thus, the HCA methodology allowed in this particular case to significantly extend the similarity between the human/yeast and malarian proteins over their whole lengths, suggesting that MAL7P1.78 can be predicted as the _Pf_TFIIA large subunit. The amino acid region separating the N- and C-terminal parts is highly variable between species [37]. In the putative _Pf _TFIIA large subunit (MAL7P1.78), this region between the N- and C-termini is shown to be smaller than in the yeast and human sequences (Fig. 2, panel A).

Using the sequence of the yeast small subunit TOA2, as query in a PSI-BLAST search, no significant sequence similarity could be found with *P. falciparum *proteins derived from the whole genome databases at convergence by iteration 2. However, a marginal similarity (*E-value of 4.6*) was highlighted with the PFL2435w hypothetical sequence, over 83 amino acids (22% identity). This similarity was supported at the 2D level using HCA (Fig. 2, panel B). It covers the N-terminal region as well as the two first strands of the C-terminal region. The third strand can be tentatively identified at the C-terminus of the *P. falciparum *sequence, when a large insertion is made between the second and third beta-strands. This large insertion likely corresponds to a globular sequence, as assessed by the presence of hydrophobic clusters. A large loop region also exists in this location in the human and yeast sequences, but was not observed in the solved corresponding three-dimensional structures. Another marginal similarity was observed at similar level of Expected-value in the PSI-BLAST results with a hypothetical protein of *P. yoelii *(PY01831; 24% identity over 82 amino acids, E-value= 0.084). However, this hypothetical protein does not correspond to the PFL2435w homolog. Instead, the PY01831 homolog in *P. falciparum *corresponds to the PFI1630c hypothetical protein (43% identity). This similarity was however not detected by PSI-BLAST because the PFI1630c sequence was incorrectly predicted (part of the coding region was inappropriately predicted as an intron; more explanations are given in the legend of Fig. 2). The corresponding alignment was also supported at the 2D level using HCA (Fig. 2; panel B). The PFI1630c hypothetical protein contains an N-terminal extension, relative to the human TFIIA γ/yeast TOA2 sequences. This suggests that two genes could exist as functional TFIIA small subunits in *Plasmodium falciparum*. Multiple genes that encode general transcription factors have already been described for the TATA-box binding protein (TBP) in several species [38-40] and for TFIIA α/β in humans [37].

### _Pf_TFIIB

TFIIB, which associates with TFIIA, is the only putative general transcription factor (PFA0525w) that was so far identified during the annotation of *P. falciparum *genome. It was confirmed by specific HMM searches performed by Coulson et al. [23].

### _Pf_TFIID

#### Evidence for the presence of some P. falciparum TBP-associated factors (TAFs) involved in the multiprotein _Pf_TFIID complex

The TATA-binding protein (TBP) and many TBP-associated factors (TAFs) form the multimeric TFIID complex [41]. While TBP is sufficient for basal transcription *in vitro*, the TAF subunits of TFIID are essential cofactors for transcriptional activation by providing interaction sites for activators. Yeast TFIID contains 14 TAFs and homologues of many of these TAFs are found in metazoans (Table 1 and [42]). Analysis of the architecture of yeast and metazoan TFIID revealed that more than half of the TAFs contains a histone fold motif (HFD) (Table 1 and [42]). These HFDs specifically assemble into five histone-like pairs.

While TBP was clearly identified in the *P. falciparum *genome (PFE0305w, Table 1), no orthologous TAFs have been described so far from the genome sequence data of several apicomplexan parasites including several *Plasmodium *species [24,43], *Cryptosporidium parvum *[44] and *T. gondii *. This suggests that this TAF detection failure cannot only be ascribed to the A+T richness of the genome. Indeed, unlike Plasmodium species, the other apicomplexan parasites do not display a bias toward A+T richness. Instead, it is likely that the amino acid sequences of TAFs in apicomplexan parasites reached a point of divergence that hinders their prediction using classical similarity searches. Here, we searched for the presence in *P. falciparum *of each of these TAFs, including those which contain histone fold motifs.

##### • _Pf_TAF1 (hTAF250/yTAF145)

**Figure 3:**
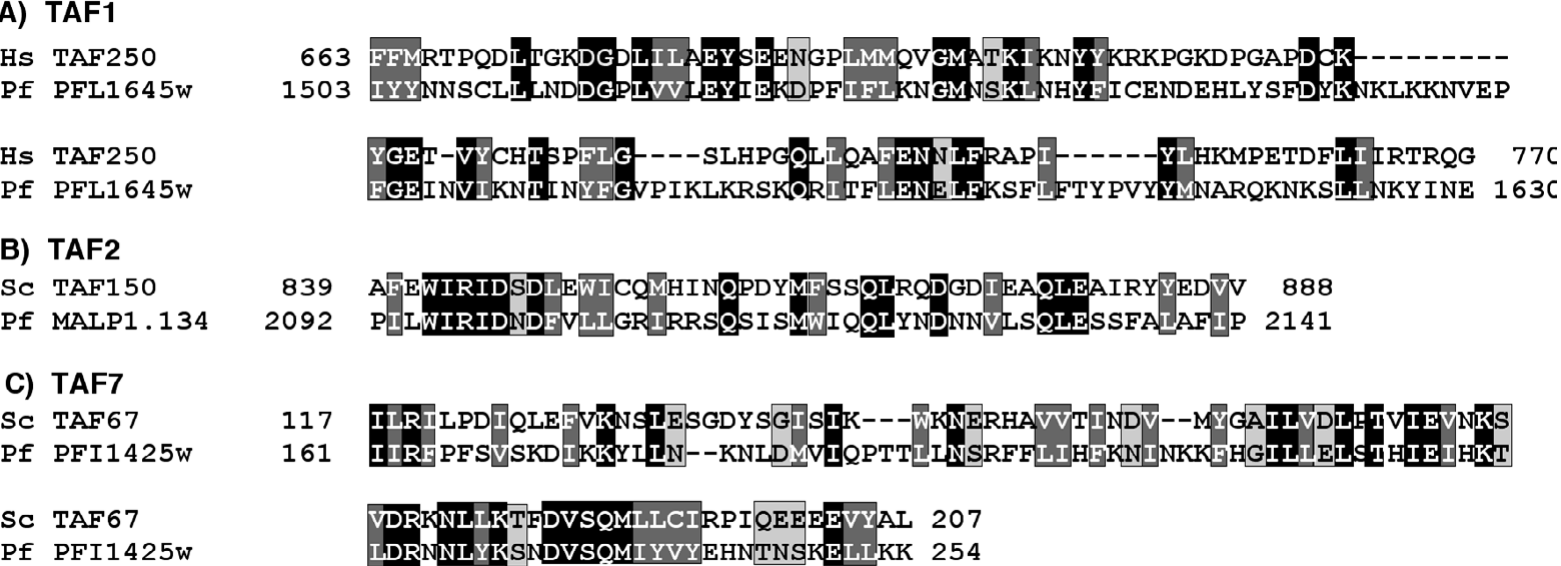
1D alignment of different TAF subunits (TAF1, TAF2 and TAF7) with hypothetical proteins from *P. falciparum*. Identical and similar amino acids are boxed in black and in grey, respectively. Although restricted to a limited length, the similarity regions highlighted here match the inter-species conserved regions (see text). These similarities are supported at the 2D level using HCA (data not shown).

In metazoan, the largest TFIID subunit has three enzymatic activities (kinase, histone acetyltransferase (HAT) and ubiquitin-activating and conjugating (ubac) activities) involved in transcriptional regulation (reviewed in [42]). Metazoan TAF250 possess a pair of C-terminal bromo domains, which recognize acetylated histones. In yeast TAF145, which otherwise lacks kinase activity, these bromodomains are not present. Instead, two interacting proteins Bdf1 and Bdf2 provide the missing enzymatic activity and functional domains. We focused our searches on the conserved domain of proteins of the TAF250 family, which is critical for HAT activity [45] (TAF1; aa 549–1290 of human TAF250). We identified, by iteration 2, a significant similarity with *P. yoelii *PY03752 (E-value 1 10^-5^), and by iteration 3 with *P. falciparum *PFL1645w (E value: 3 10^-13^) (Fig. 3, panel A). These similarities were supported at the 2D level using HCA (data not shown). These two orthologous hypothetical proteins possess one bromo domain in their C-terminal parts. Careful study of their HCA plots led to define the limits of the conserved domain between aa 1420 and 1650 (PFL1645w). Clear hinge regions could also be identified. Using this domain as query in PSI-BLAST led to the identification of all members of the TAF250 family by iteration 4. No other protein from the *P. falciparum *genome data was identified, suggesting that PFL1645w might be the genuine TAF1 ortholog.

###### • _Pf_TAF2 (hTAF150/yTAF150)

TAF150 proteins have a non-specific aminopeptidase domain in their N-terminal parts. We therefore focused our searches on the C-terminal parts of the proteins. Using PSI-BLAST and the yeast TAF150 C-terminal domain (aa 701 to 1407) as query, a significant hit appeared by iteration 2 with the *P. yoelii *PY03343 hypothetical protein (E-value 0.002), together with those relative to other metazoan TAF150. The identification of *P. yoelii *orthologous TAF2 has been used to discover the *P. falciparum *TAF2, which is currently named in the annotated genome as the hypothetical protein MAL7P1.134. This sequence was scored with a significant E-value (3 10^-7^) by iteration 3 (Fig. 3, panel B). This similarity is supported at the 2D level and concerns the region which is most conserved in the TAF150 C-terminal domain amongst the different species. This suggests that the *P. falciparum *protein pinpointed here might be the TAF2 ortholog in the parasite.

###### • _Pf_TAF7 (hTAF55/yTAF67)

TAF7 proteins possess a conserved domain (TAFII55 protein conserved region), located between amino acids 112 and 305 (yeast) or amino acids 12 and 178 (human) [46]. Using this domain as query in PSI-BLAST led to the identification of significant similarities from the second iteration with both *P. yoelii *PY04173 (E-value 2 10^-6^) and *P. falciparum *PFI1425w (E-value 2 10^-6^) hypothetical proteins. This similarity, limited to the first part of the TAFII55 protein conserved region (PFI1425w aa 161 to 242), is supported at the 2D level (Fig. 3, panel C). This similarity was also retrieved when scanning the Pfam database (pfam04658.5, TAFII55_N). However, the globular domain of the *P. falciparum *proteins in which the TAFII55-like region is included appears to be larger (aa 148 to ~ 325), and thus might share a similar length to the complete TAFII55 protein conserved region. The region of similarity shared by *P. falciparum *PFI1425w and other TAF7 was previously shown to be critical for interaction with the bromo domain factor Bdf1 of yeast cells [46].

###### • _Pf_TAF10 and the apparent lack of TAFs assembling into histone-like pairs in P. falciparum

The histone fold domain (HFD), the core of which is characterized by three alpha-helices, is a fundamental interaction motif involved in heterodimerization of the core histone (H4-H3, H2A-H2B) and their assembly into a nucleosome octamer. This motif is thought to have arisen from the duplication of a minimal helix-extended-helix structure. The two middle helices of the duplicated structure would have fused to form a long, central helix. The histone fold domain can be accompanied by N- or C-terminal extensions, also made of alpha-helices and is found in several non-histone proteins, in addition to core histones [42,47].

Analysis of TFIID has shown that more than half of the TAFs constituting this complex are HFD containing proteins (reviewed in [42]). This led to the first hypothesis of a compact nucleosome-like octamer core in TFIID, which could bind DNA and around which other TAFs could associate [48] (reviewed in [49,50]). This proposal has however to be revisited in light of recent experimental data, highlighting a more complex situation than initially thought. First, irrespective to the nature of the quaternary structure (nucleosome-like octamers, as observed for the TAF4/TAF12 – TAF6/TAF9 assembly [51], or other structures), it has been shown that surface residues of core histones known to make critical contacts with DNA in the nucleosome are generally not conserved in TAF HFDs [52,53]. This suggests an alternative role for HFD in TAFs than DNA binding. Second, immunolabeling electron microscopy experiments have demonstrated that the HFD-containing TAFs are located in three distinct substructures of TFIID, which are assembled by thin linker domains in a molecular clamp architecture [54]. The TAF4/TAF12 – TAF6/TAF9 assembly was shown to co-localize in one of the three lobes of native TFIID [55]. These structural data were supported and enriched by additional mapping of other TFIID functional sites [55].

Our searches for HFD-containing TAFs in the *Plasmodium *genome did not lead to the identification of any of the five histone-like pairs currently known in other eukaryotic species (TAF6-9, TAF11-13, TAF4-12, TAF3-10 and TAF8-10). These searches were performed using as queries the full-length sequences of yeast and human TAFs, their HFDs, and specific domains accompanying HFDs (*e.g. *for TAF4, we considered the specific TAF4 domain, including HFD (hTAF135 aa 832 to 1083); the HFD (hTAF135 aa 835 to 950) and the TAFH sequence (hTAF135 TAF homology region, also known as nervy homology region 1 (NHR1); smart00549; aa 590 to 649)).

**Figure 4:**
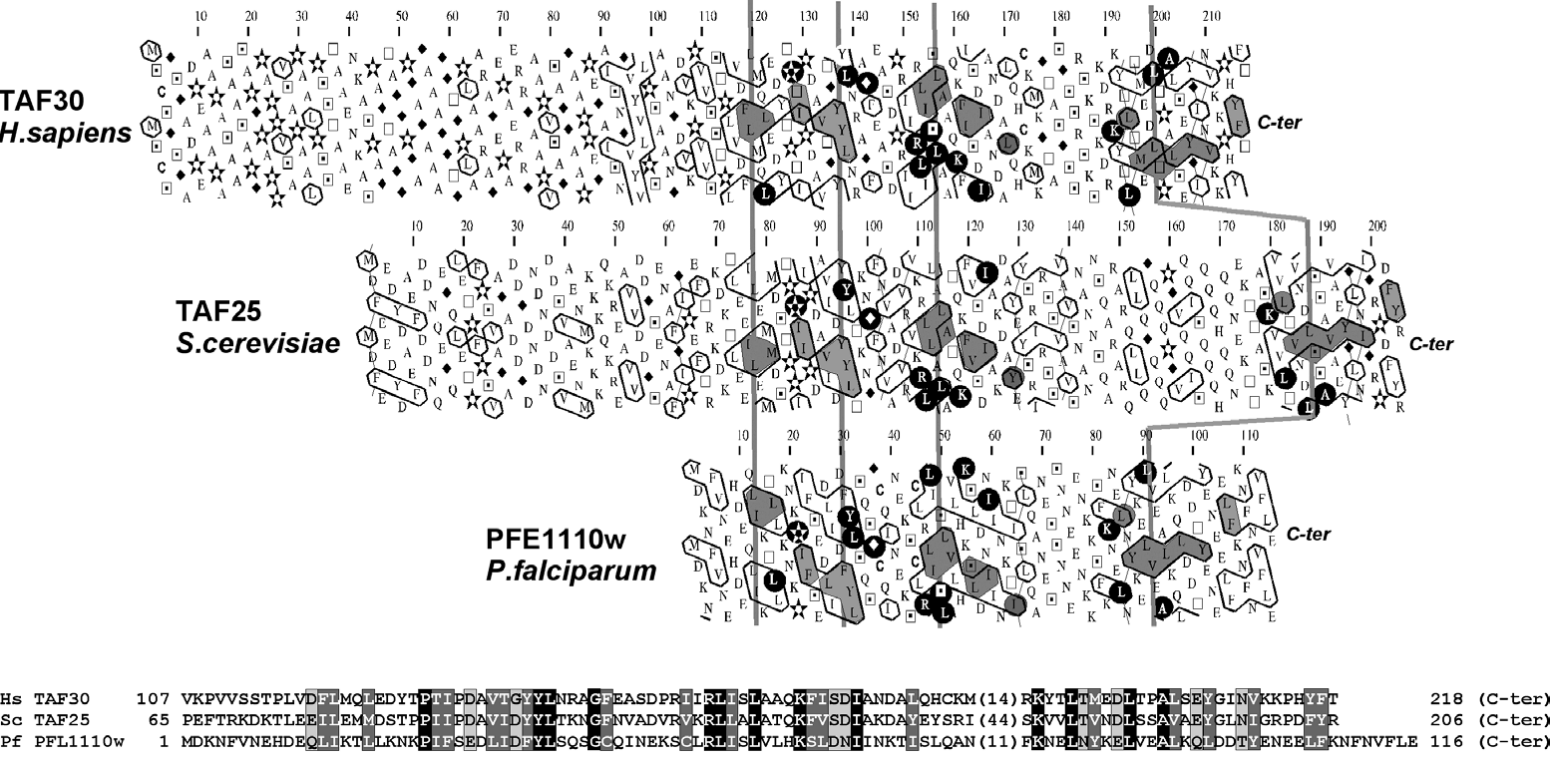
Comparison of the HCA plots of TAF10 from human (TAF30) and yeast (TAF25) and the *P. falciparum *hypothetical protein PFE1110w (see figure Fig. 2 for explanation) Cluster similarities are shaded in grey, identities are shown in white on a black background. Vertical bars indicate cluster links. The deduced 1D alignment is shown at the bottom.

Only one candidate for TAF10 was retrieved in the *Plasmodium *genome. The TAF10 subunit in yeast, TAF25, heterodimerizes with TAF3 (yeast TAF47) and TAF8 (yeast TAF65) [50]. It is one of the essential component common to TFIID and SAGA [56]. Yeast TAF25 was predicted to have a HFD, with which it can dimerize with its partner [50]. However, the presence of HFD in yeast TAF25 has not yet been experimentally demonstrated. Searching databases using the yeast TAF25 sequence as a probe led to the identification by convergence of marginal similarities with the *P. falciparum *PFE1110w hypothetical protein (E-value 1.5; 25% sequence identity over 52 amino acids). These similarities were supported at the 2D level (Fig. 4). A reciprocal search yielded the TAF10 proteins as the first hits above the threshold value.

Taken together, our data strongly suggest the apparent and unexpected lack of HFD containing TAFs in *P. falciparum*, except from TAF10. This TAF however remains to be determined as a genuine HFD-containing factor in the parasite and also in the other eukaryotic cells.

#### Other undetected TAFs within the multiprotein PfTFIID complex

##### • _Pf_TAF5 (hTAF100/yTAF90)

This protein, interacting with TFIIFβ, contains WD40 repeats. We specifically limited our searches to the WD40 associated region in TFIID subunit (pfam04494; aa 194–340 of hTAF100), but these did not lead to the identification of potential TAF5 candidates. We were thus unable to report the presence of a putative TAF5 ortholog in *P. falciparum*.

##### • _Pf_TAF14 (hENL-AF9/yTAF30)

**Figure 5:**
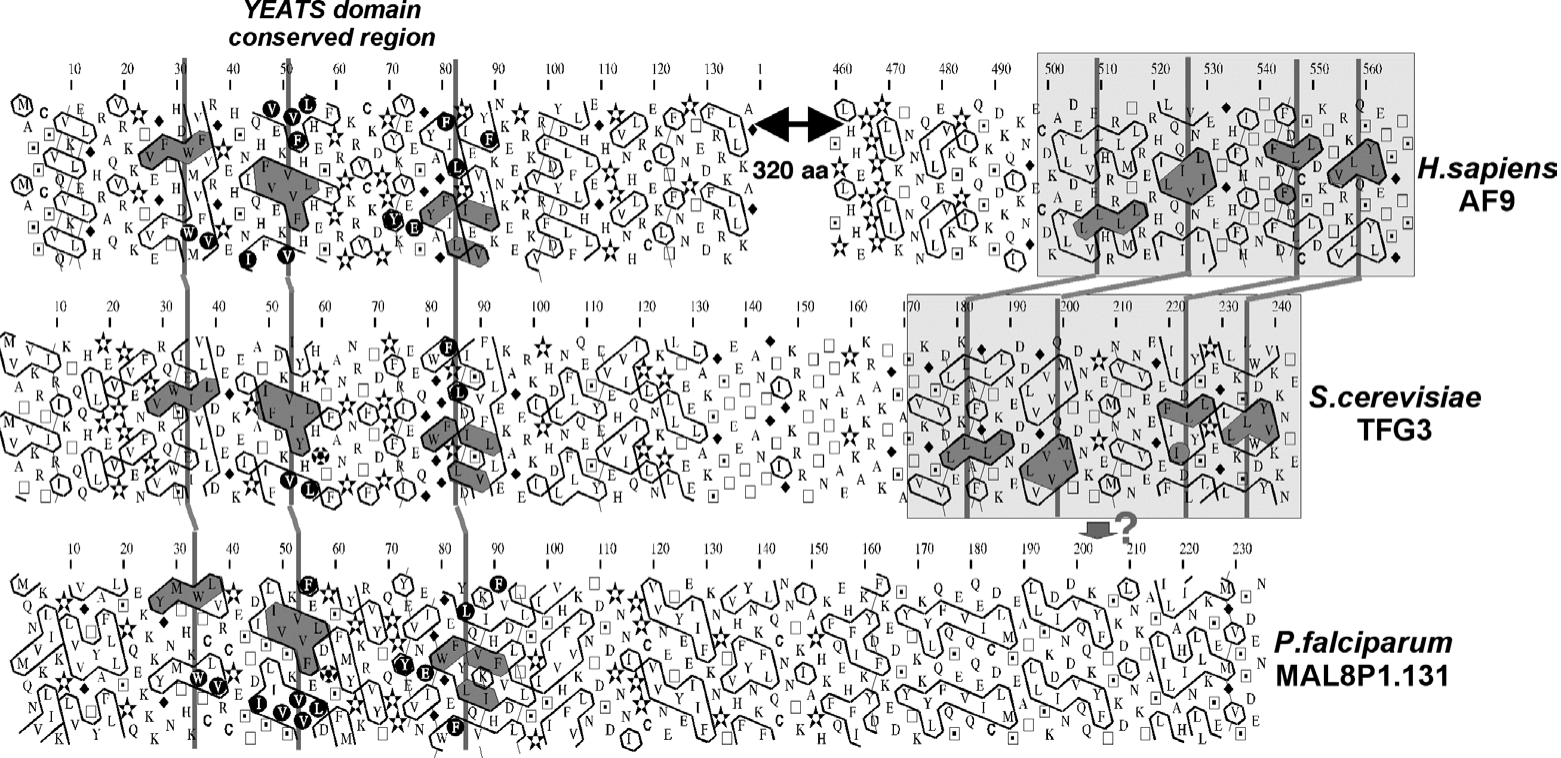
Comparison of the HCA plots of the TAF14 subunit in humans (AF9) and in yeast (TFG3) with that of the *Plasmodium falciparum *MAL8P1.131 hypothetical protein (see Fig. 2 for explanation). Cluster similarities are shaded in grey and identities are shown in white on a black background. A N-terminal YEATS domain [57] is present in the three sequences, whereas HCA detects a common domain in the C-terminal end of only human AF9 and yeast TFG3. The conserved clusters of this C-terminal domain are not detected in the MAL8P1.131 sequence, suggesting that this protein may not correspond to _Pf_TAF14.

TAF14, named AF9 in human and TAF30 in yeast, is the only non-essential TAF. It is also a component of TFIIF in yeast. TAF14 contains two globular domains, the N-terminal one belonging to the YEATS family of domains, which are found in several proteins involved in chromatin modification and transcriptional regulation [57]. A YEATS protein can be found in the *P. falciparum *MAL8P1.131 protein, described as a Gas41 homologue. However, the *P. falciparum *MAL8P1.131 protein does not possess the C-terminal domain common to yTAF30 and hAF9. The similarity between the C-terminal domains of these proteins can be only detected using HCA (Figure 5). However, the clusters typifying the architecture of the C-terminal TAF14 domain are not found in MAL8P1.131 protein, suggesting that this protein may not correspond to a genuine _Pf_TAF14.

### _Pf_TFIIE

**Figure 6:**
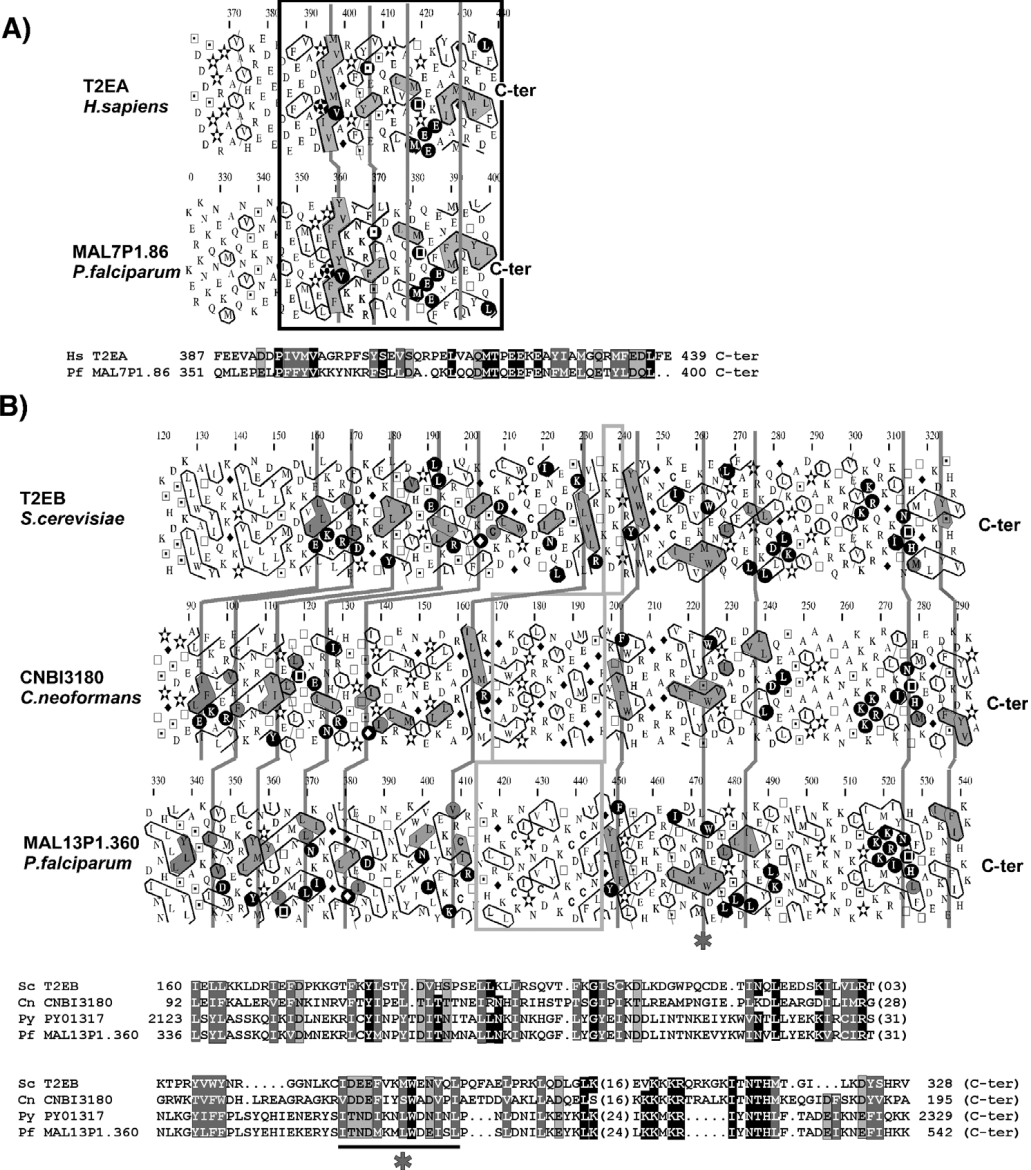
A) Identification of a small globular domain common to the C-termini of TFIIE α subunits of higher eukaryotes and *P. falciparum*. Top panel: Comparison of the corresponding HCA plots (see figure Fig. 2 for explanation). Cluster similarities are shaded in grey and identities are shown in white on a black background. The position of the globular domain is boxed. Despite the low level of sequence identity, hydrophobic clusters are well conserved, supporting the presence of a common fold. Bottom panel: HCA-deduced 1D alignment. B) Comparison of the TFIIE β subunits of *S. cerevisiae*, *Cryptococcus neoformans *and *Plasmodia *species (*P. yoelii *and *P. falciparum*). Cluster similarities are shaded in grey and identities are shown in white on a black background. The deduced 1D alignment is shown at the bottom.

TFIIE is an heterotetramer composed of a large and a small subunit, referred as α (human)/ TFA1 (yeast) and (human)/ TFA2 (yeast), respectively [58,59]. A clear homologue of the TFIIE α subunit, the MAL7P1.86 hypothetical sequence, was readily identified from the first iteration starting from human TFIIE α sequence (*E-value *1 10^-5^; sequence identity of 26% over the first 162 amino acids of TFIIE α totalizing 439 amino acids). This TFIIE α homologue was also reported by Coulson et al. [23] and has recently been designated in sequence databases as the putative TFIIE α-subunit. The N-terminal sequence pinpointed here is required for activation of basal transcription *in vitro *through interactions with TBP and Pol II [60,61]. This N-terminal sequence contains an extended winged helix domain [62] followed by a zing finger [63]. Most importantly, the analysis reported here led to extend the similarity between the human and *P. falciparum *proteins over a small C-terminal globular domain of TFIIE α, as secondary structure similarities, supported by sequence identities, could be clearly identified (Fig. 6, panel A). This similarity was not detected by PSI-BLAST (below and up to the threshold *E-value*), even if only this small domain instead of the whole protein was used for similarity searches. The small acidic globular domain, for which no known fold could be predicted by threading procedures (3D-PSSM, [64]) (data not shown), is absent in the yeast sequence, the C-terminal of which is likely unstructured [65]. This domain may thus be specific of higher eukaryotes and *P. falciparum*. This region binds directly to TFIIH and has a stimulatory effect on basal transcription [61].

No *P. falciparum *orthologue could be found for the second subunit of TFIIE, named TFIIE  in a first approach using as queries the human and yeast sequences in PSI-BLAST searches. These two sequences display a low level of sequence identity (less than 30%), especially concentrated in the C-terminal segment. We adopted an iterative strategy, by using distant sequences of the family described in the PSI-BLAST data and thereby discovered potential relationships with *Plasmodium *proteins. Hence, using the sequence of the hypothetical protein CNBI3180 from *Cryptococcus neoformans*, which shares 22% of sequence identity with the *S. cerevisiae *TFA2 over 293 amino acids (*E-value *by convergence 1 10^-28^), we found by iteration 3, a significant similarity with the C-terminal fragment of a hypothetical sequence from *P. yoelii *(PY01317; 15% identity over 219 amino acids, E-value 2 10^-38^). This relationship was supported at the 2D level (Fig. 6, panel B), especially highlighting well conserved hydrophobic clusters common to distant members of the family (for example, see the hydrophobic cluster highlighted with an asterisk in Fig. 6, panel B). The corresponding sequence in *P. falciparum*, MAL13P1.360, was found by searching the predicted annotated proteins within PlasmoDB (version 4.3, November 2, 2004; 81% sequence identity with PY01317). The comparison of the whole set of sequences of the TFIIE β subunit family revealed a region of variable length in the middle of the TFIIE β domain. This region is particularly rich in cysteine residues in the proteins of apicomplexan parasites such as *P. falciparum*, *P. yoelii and Cryptosporidium *(boxed in Fig. 6, panel B).

### _Pf_TFIIF

TFIIF, a tetramer of two subunits, named α (mammalian RAP74/yeast TFG1) and β (mammalian RAP30/yeast TFG2), is intimately associated with the RNA polymerase II enzyme [66]. The TFIIF complex directly binds promoter DNA, TFIIB and the TAF250 subunit of TFIID, and recruits TFIIE and TFIIH to the preinitiation complex [67]

**Figure 7:**
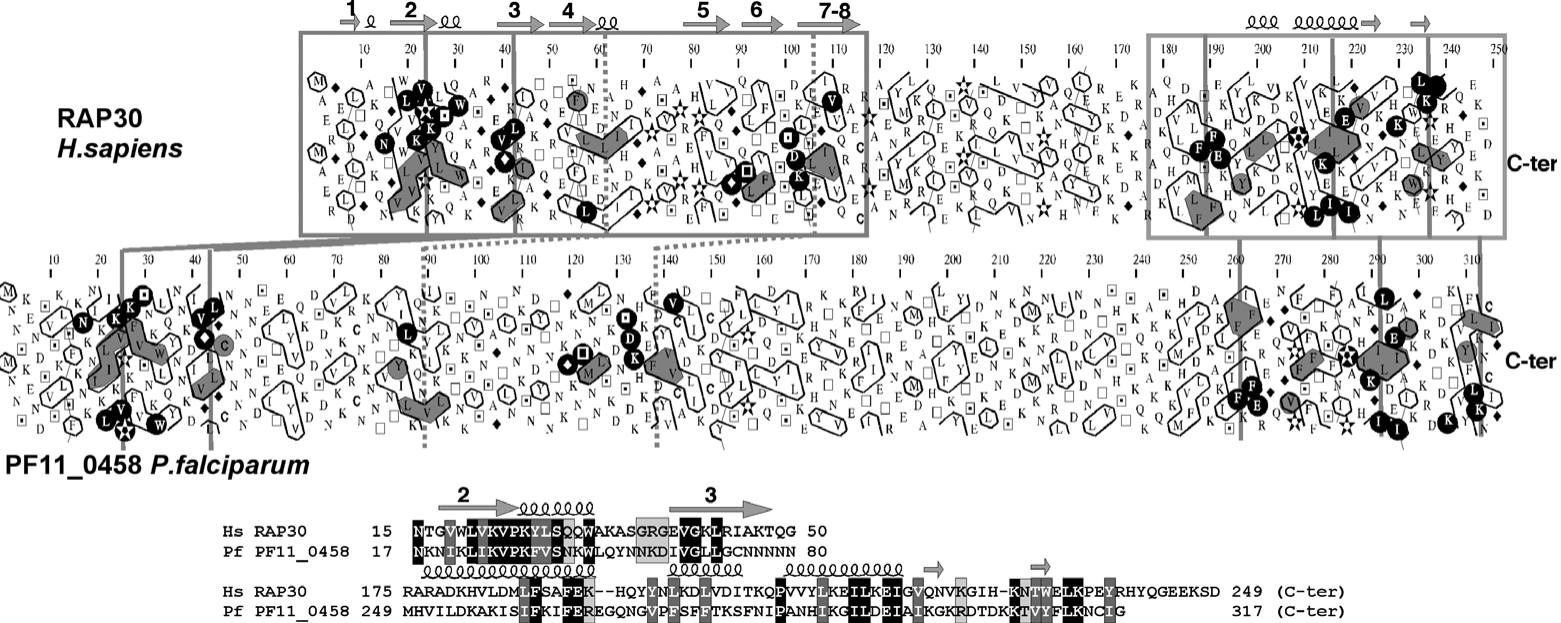
Comparison of the human TFIIF β subunit (RAP30) with the hypothetical protein PF11_0458 from *P. falciparum*. Top panel: Comparison of the corresponding HCA plots (see Fig. 2 for explanation). The N- and C-terminal structured domains are boxed, according to the limits defined on the basis of experimental data (pdb 1f3u (chain A) and 2bby, respectively). Cluster similarities are shaded in grey and identities are shown in white on a black background. Putative correspondences in the C-terminus of the first domain are reported with dashed lines. Secondary structures, as observed in the experimental structures, are reported up to the RAP30 sequence. The corresponding 1D alignment is shown in the bottom panel.

The β-subunit can be divided into two globular regions separated by a central, less structured region. The structures of these two globular domains have been solved in human RAP30 [68,69]. The N-terminal domain is responsible for RAP74 and TFIIB binding [68], and forms with the N-terminal domain of RAP74 a triple barrel dimerization fold [70]. The C-terminal domain, containing a winged-helix [69], binds non-specifically to DNA [71]. Using the human RAP30 and yeast TFG2 sequences as queries in PSI-BLAST, no significant similarities with *P. falciparum *proteins were detected at convergence by iteration 3. However, marginal similarities were detected above the threshold value with the hypothetical PF11_0458 protein (E values 9.4 and 0.25, respectively; 12% and 19% identity over 326 and 324 amino acids, respectively). These similarities were supported at the 2D level (Fig. 7). Some of the regular secondary structures constituting the core of the globular domains are particularly well conserved, whereas the region separating these domains appears less similar, as observed for other β subunits of different species. However, a good conservation was observed only for the strands beta-2, beta-3 and beta-4 of the N-terminal domain, suggesting that the structure of the parasite protein may locally differ from that of human RAP30.

RAP74 (α subunit) also possesses two N- and C-terminal globular domains, separated by an unstructured linker sequence. As indicated above, the N-terminal domain, which is responsible for TAF250 and RAP30 binding, forms with the RAP30 N-terminal domain a triple barrel dimerization fold [70]. The C-terminal domain, interacting with TFIIB, FCP1 and DNA, folds as a winged-helix [72]. We thus restricted our queries to these two domains, the limits of which were identified through HCA (from aa 1 to 180 and 450 to end). However, we found no significant similarity with any parasite proteins including several *Plasmodium *species and *Cryptosporidum parvum*. We also did not see any marginal similarity, which could be confirmed at the 2D level. Given the single core structure that the two subunits form together, with three interwoven beta- barrels, it seems unlikely that the RAP30 homolog exists alone in apicomplexan parasites. Alternatively, either the RAP74 subunit is too divergent to be detected using the available tools, including HCA, or it does not really exist, suggesting the replacement of a α-β heterodimer by a β-β homodimer instead, given the similar architecture of the two subunits.

### _Pf_TFIIH

The general transcription factor TFIIH is the largest and most complex of all. Indeed, it is composed of nine subunits with molecular mass (460 kDa) similar to that of RNA PII, with several subunits having enzymatic activities (reviewed in [67,73-75]). TFIIH has a dual action in both transcription initiation and nucleotide excision repair (NER). It is organized into two structural and functional entities. The first of these, the TFIIH core, includes four polypeptides (named P62, P52, P44 and P34 in human; yeast orthologous sequences are indicated in Table 1) and the xeroderma pigmentosum B (XPB) helicase. The second functional entity, the CDK-Activating Kinase (CAK) complex, is composed of the cyclin-dependent kinase Cdk7, cyclin H and MAT1. The XPD (RAD3) helicase bridges the two complexes, being associated either with the core or CAK. In addition to this, a new subunit of the TFIIH core, TFB5, has recently been discovered, associated in humans with DNA repair-deficient trichothiodystrophy [76,77].

**Figure 8:**
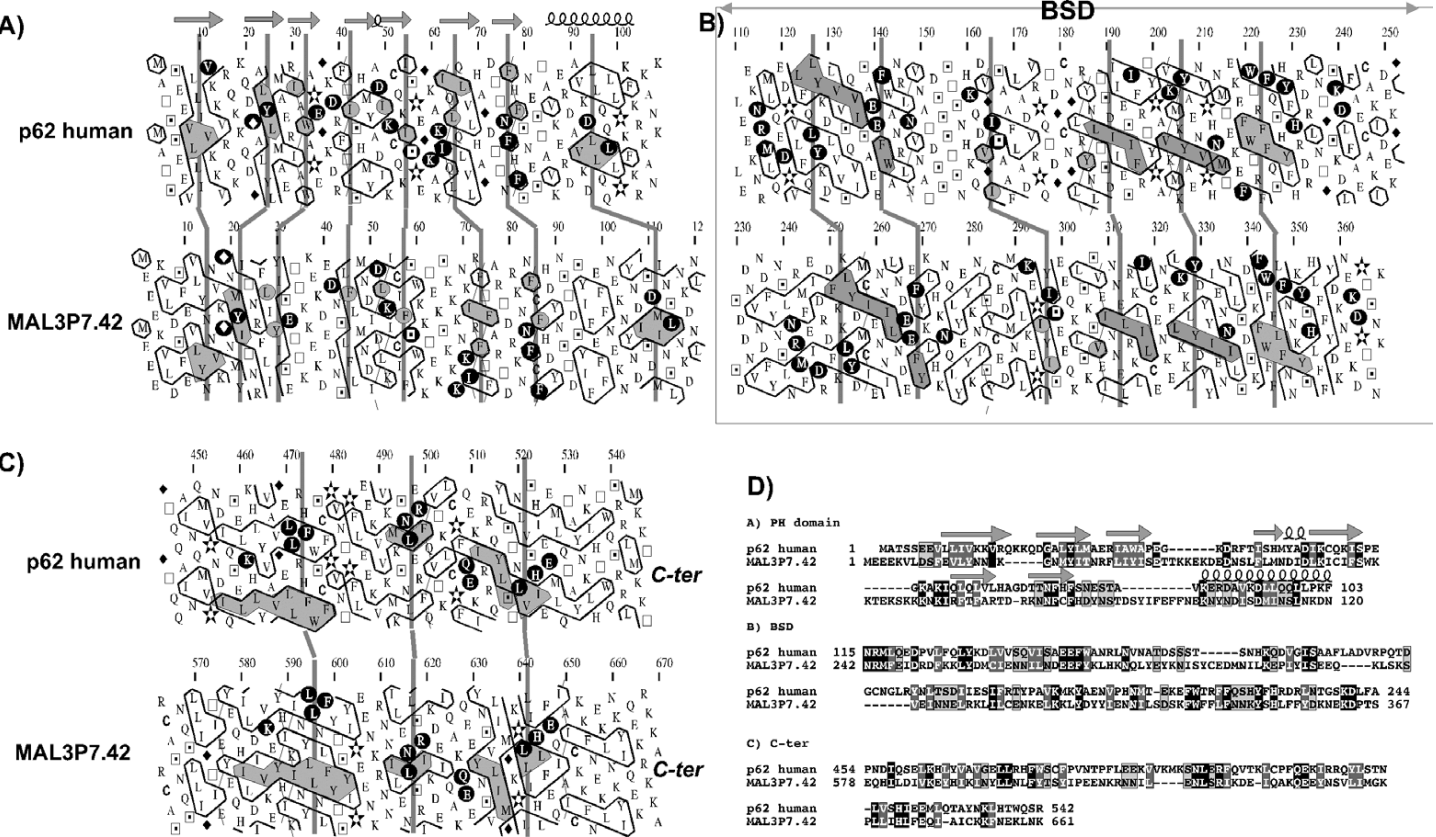
Comparison of the human TFIIH P62 subunit with the hypothetical MAL3P7.42 from *P. falciparum*. Significant similarities were detected for the region including two BSD domains (boxed, panel B). These were supported at the 2D level by comparison of the HCA plots (see Fig. 2 for explanation), where cluster similarities are shaded in grey and identities are shown in white on a black background. Marginal similarities observed in PSI-BLAST are located within the C-terminal parts of the proteins (ranging from ~250 to the C-terminus (human) and from ~aa 370 to the C-terminus (*P. falciparum*)). These were also supported at the 2D level, as illustrated here on a segment in the most distal C-terminal part of the two proteins (panel C). Upstream of the BSD domain, cluster similarities together with sequence identities can be observed in the most proximal N-terminal part of the protein sequences, corresponding to a pH domain in human p62 (panel A). Secondary structures, as observed in the experimental structure of human p62 (pdb 1pfj; [80]), are reported up to its sequence. The corresponding 1D alignment is shown in panel D.

TFIIH is the most thoroughly documented complex in *Plasmodium falciparum*. Indeed, five out of six core subunits (except from human P62/ yeast TFB1) can easily been identified using profile-based searches. The XPD (RAD3) helicase and components of the CAK complex [23] were also identified (Table 1). However, no direct homologue of Cdk7 and Cyclin H, sharing a high level of sequence identity with the human and yeast counterparts, was identified. Nevertheless, several Cdk7 and Cyclin H putative homologs with lower identity values can be identified. However, their exact nature remains to be determined. Coulson et al. [23] noticed the presence of a cyclin K homolog (PF13_0022), which was shown to be associated with RNAP II [78]. We focused our searches on the missing P62/TFB1 subunit, which possesses two copies of a BSD domain [79] and was recently shown to possess in its N-terminus a domain with a PH fold [80]. *Plasmodium *sequences marginal similarities were detected by searching databases using the human p62 as query. The sequence alignments display E-values of 0.35 with *Plasmodium yoelii *PY00359 and 10 with *Plasmodium falciparum *MAL3P7.42. The corresponding similarities were supported at the 2D level (Fig. 8, panels B and D). The similarities with these *Plasmodium *sequences were found to be significant when the yeast TFB1 sequence was used instead as query (E-values after convergence by iteration 6 of 2 10^-68 ^and 2 10^-59 ^for the alignments with the PY00359 and MAL3P7.42 sequences, respectively – 14% and 13% identity over 430 and 432 amino acids, respectively). Most of the similarities were focused on the BSD domains (Fig. 8, panels B and D), but longer alignments within the C-terminal part of the sequences were also observed. These C-terminal alignments were also supported at the 2D level, as illustrated in Fig. 8 panel C, and include sequences which are predicted to form long helical structures. Finally, HCA suggests that the N-terminal sequence of P62/TFB1, corresponding to the PH domain [80], might also be aligned with the *Plasmodium *sequences, although this relationship was not highlighted using PSI-BLAST (Fig. 8, panel A).

## Discussion

Only a few components of the general transcription machinery have been identified to date in *P. falciparum *[23]. Of the 33 general transcription factors listed in Table 1, only one third (ten subunits) were predicted from simple similarity searches [24] and previous analysis [23]. This percentage may reflect the poor proportion of gene with automatically predicted function in the complete parasite genome [24]. Hence, the TATA binding protein or TBP is the only known component of the TFIID complex, which has been identified. The multicomplex TFIID remains however essential for accurate and higher transcription levels in eukaryotic cells. Therefore, the paucity of both defined malarial TFIID orthologous components and of general transcriptional factors, contrasts significantly with the situation reported for the crown group eukaryotes, in which TFIID is well conserved even though some differences can be seen for transcription cofactor complexes. Here, the use of the sensitive Hydrophobic Cluster Analysis (HCA) in combination with profile-based search methods suggests that the genome of *P. falciparum *contains several gene products annotated as hypothetical proteins, which can be predicted as putative general transcription factors (GTF) associated with RNAP II. These include several members of TFIID even if most of the TAF containing histone fold domains (HFDs) remain undetected using the sensitive Hydrophobic Cluster Analysis (HCA). Nine other GTFs were predicted in this way, which brings the total number of predicted subunits of the general transcription machinery to approximately 60% that of the number observed in the crown group eukaryotes.

**Figure 9:**
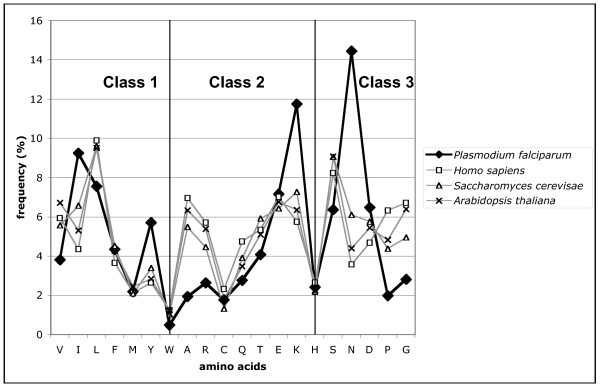
Comparison of the amino acid distribution in the proteomes (predicted proteins) of *P. falciparum*, *Homo sapiens*, *Saccharomyces cerevisiae *and *Arabidopsis thaliana *(5334, 32035, 6699 and 27857 sequences, respectively). Amino acids are grouped with respect to the structural classes defined previously in [30]. The first class defines strong hydrophobic amino acids (V, I, L, F, M, Y, W), that display a similar propensity for yielding regular secondary structures (α-helices and β-strands. The third class includes coil-forming amino acids (G, P, D, N, S), whereas the intermediate class (A, R, C, Q, T, E, K) contains amino acids for which coil and secondary structure forming propensities are similar. The total class I amino acid content is similar in *Plasmodium falciparum *with respect to other proteomes (see comments in the discussion section). The frequency of cysteine, which is also a frequent component of hydrophobic cores, does not differ in *Plasmodium falciparum *relatively to other organisms. One can also observe a stable frequency for histidine, which has always a remarkably neutral behavior in the secondary structure propensities.

The lack of detection of several GTFs in the parasite using conventional methods for sequence similarity searches could be ascribed to a higher divergence of these proteins, as well as to the bias introduced in the searches by the overall high A+T nucleotide content. The apparent divergence between *Plasmodium *GTFs relative to their orthologs in free-living organisms is consistent with the observation previously reported by Coulson et al. [23,81]. These authors have noticed that transcription-associated proteins family taxon specificity appeared to correlate with evolutionary distance and not cellular complexity. On the other hand, *Plasmodium *sequences are very difficult to analyze due to a particular amino acid bias, reflecting the overall high A+T nucleotide content. This results in unusually high proportions of asparagine and lysine, and to a lesser extent also of isoleucine and tyrosine, which are all encoded by AT-rich codons. This abundance contrasts with a relative paucity in arginine, alanine, proline and glycine, encoded by GC-rich codons (Fig. 9 and [82]). However, part of the most abundant amino acids, asparagine and lysine, is located within the low complexity regions that are often located outside functional domains (K. Prat, J.P. Mornon and I. Callebaut, unpublished results). When we evaluated the presence of the hydrophobic core forming amino acids (class 1, Fig. 9) which are crucial for fold stability, the *P. falciparum *proteome is similar to the other organisms for the frequencies of phenylalanine, methionine and tryptophane. In addition, there is a nearly perfect balance between valine, leucine and isoleucine, which are chemically related and often interchangeable at the structural level. The last amino acid of the hydrophobic class, tyrosine, is the most coil-forming residue of this class [30] and the increase of its frequency in *P. falciparum *may actually not affect the general balance of domain hydrophobic cores. The high frequency of lysine, which is on average the most exposed amino acid in globular domains [83-85], might be balanced by those of arginine and alanine. Within the third class (coil-forming residues), the low frequencies of proline and glycine, which have on average a similar behavior relative to α, β and coil states [32], might be together compensated by the considerable high frequency of asparagine residues. Asparagine immediately after glycine, shares with it the ability to adopt left-handed helical local conformations, widely represented in coil regions. Thus, the conservation of the total proportion of hydrophobic amino acids in *P. falciparum*, and the compensative behavior of other amino acid couples, gives evidence for the conservation of hydrophobic cores of functional domains. This also suggests that an appropriate delineation of these domains through HCA, and their specific use for sequence similarity searches, can lead to the finding of significant relationships within proteins, which otherwise remain orphan if their sequences are analyzed as a whole. The development of an automatic procedure allowing a fast prediction of the structured domain boundaries can help to apply such a strategy at the genome-scale level (K. Prat, J.P. Mornon and I. Callebaut., unpublished results).

A first original feature in the *P. falciparum *predicted GTF sequences is the presence of two genes candidates for the TFIIA small subunit. The presence of two genes has already been described for some GTFs. Indeed, TBP-like proteins are found in *A. thaliana*, *D. melanogaster *and *H. sapiens *[38-40]. Moreover, a functional homolog of TFIIA α/β subunit, which is expressed almost exclusively in testis, has been described [37]. This multiplicity of GTFs is thought to contribute to tissue- and gene-specific regulation. It is therefore possible that the gene candidates for the TFIIA small subunit in *P. falciparum *are stage-specifically expressed in the parasite life cycle.

Another striking observation is that, among the GTFs associated with RNAP II predicted here, no HFD-containing TAFs could be identified, except for the putative ortholog of TAF10, which was reported by Gangloff and colleagues [50] as containing a potential HFD. However, the potential HFD of TAF10 might correspond to a distant member of the HFD family and remains to be experimentally proven. On the other hand, TAF10 is the only "HFD" protein which is shared by TFIID and SAGA [56]. It is interesting to note the apparent lack of TAF10 candidate in *P. yoelii *(Table 2), suggesting that this protein might not be essential in all *Plasmodium *species. This may be consistent with the apparent absence of other HFD-containing TAFs in all *Plasmodium *species. The apparent absence of canonical HFD TAFs leads to the hypothesis of a higher divergence of proteins of the HFD family in the *Plasmodium *genome than that of other proteins, beyond the point where they can be identified using homology searches, even the most sensitive of them. Alternatively, if the absence of HFD containing TAFs is confirmed, this will provide evidence for a striking difference in the quaternary structure of TFIID by comparison to the yeast or human complexes. The latter display a similar architecture, formed by three lobes organized into a molecular clamp [54,86,87]. Experimental investigations are needed to further explore this hypothesis. To date, the only *Plasmodium *proteins with HFD domains listed in the histone database [47] corresponds to classic nucleosomal histones H2A, H2B, H3 and H4. The linker histone H1 is not found [22]. This suggests that the apparent absence of histone fold proteins in *Plasmodium *is not only restricted to the TAF proteins of TFIID complex.

## Conclusion

In conclusion, we have shown that more general transcription factors can be predicted in the genome of *P. falciparum *than initially thought. It can be anticipated that the HCA method can also be an additional and important tool for the finding of new orthologs amongst the high proportion of hypothetical proteins or orphans in *P. falciparum *and other apicomplexan parasites such as *Cryptosporidium parvum*, *Eimeria *and *Toxoplasma gondii*. Virtually nothing is known about transcription regulation in these apicomplexan parasites. To our knowledge, this study describes for the first time the prediction of general transcription factors in the genome of *P. falciparum *using a sensitive predictive method based on secondary structure considerations (HCA). Based on the GTF orthologs predicted here, there are some differences in the composition, and probably in the nature of some multicomplex factors, as illustrated by the possible absence of HFD containing TAFs in the TFIID complex. The identification of novel transcription elements and understanding how the basal transcription differs in the parasite may be exploited to design selective therapeutic agents against *P. falciparum*. Additionally, further elucidation of mechanisms controlling transcriptional expression in protozoa may provide a unique perspective on how these systems evolved in early eukaryotic cells.

## Methods

The non-redundant database (NR; 2 456 374 protein sequences at May 3, 2005) at NCBI (National Center for Biological Information) was searched using the BLASTP program with default parameters [88] (BlastP 2.2.10, Oct 19, 2004; Blosum 62, gap penalties: existence 11, extension 1). Profile searches were conducted using PSI-BLAST, run until convergence with a default profile inclusion expect (E) value threshold of 0.005. Reciprocal searches were carried out for all the predicted GTF components of *Plasmodium falciparum *(see comments in the Result sections). The PlasmoDB (version 4.3, November 2, 2004) [25,89] was also searched using the same tools (BLASTP 2.1.2). Other databases (Pfam [90], Smart [91]; CDD [92]) were also searched for the presence of known domains.

The two-dimensional Hydrophobic Cluster Analysis (HCA) [29,30] was used to sort at the two-dimension level (2D) the potential sequence and structure relationships. HCA offers the possibility to add to a literal analysis, a lexical one by identifying the regular secondary structures from the consideration of a single sequence. Indeed, the positions of hydrophobic clusters were shown to mainly correspond to the positions of regular secondary structures [31]. These non-intertwined binary patterns, constrained by the consideration of a connectivity distance separating two distinct clusters on the two-dimensional plot (the currently used alpha-helix is associated with a connectivity distance of 4), are much more informative than non constrained ones [32]. Hence, similar structures are often associated with conservation of hydrophobic cluster features, which participate in the protein core, together with sequence similarities. This conservation often helps or allows the alignment procedure for highly divergent sequences (typically within and below the twilight level). This approach has been used to identify new domains (e.g. [93,94]), link orphan sequences to structural and functional families (e.g.[95,96]) or identify and characterize catalytic sites (e.g. [97-99]). Other examples can be found at [100].

## Authors' contributions

IC and ST conceived the study and drafted the manuscript. IC carried out the sequence analysis, in which EM and JPM participated. KP performed the statistical analysis of genome sequences.
